# Quality of cognitive-behavioural therapy in routine psychiatric care: therapist adherence and competence, and patient outcomes for depression and anxiety disorders

**DOI:** 10.1186/s12888-024-06328-4

**Published:** 2024-12-04

**Authors:** Hillevi Bergvall, Johanna Linde, Sven Alfonsson, Rikard Sunnhed, Jacques P. Barber, Tobias Lundgren, Gerhard Andersson, Benjamin Bohman

**Affiliations:** 1 Department of Clinical Neuroscience, Centre for Psychiatry Research, Karolinska Institutet and Stockholm Health Care Services, Region Stockholm, Stockholm, Sweden; 2 Gordon F. Derner School of Psychology, Adelphi University, Garden City, NY USA; 3 Department of Behavioural Sciences and Learning, Linköping University, Linköping, Sweden; 4 Department of Biomedical and Clinical Sciences, Linköping University, Linköping, Sweden

**Keywords:** Cognitive behavioral therapy, Clinical competence, Depressive disorder, Anxiety disorders, Health care quality assessment

## Abstract

**Background:**

Quality of care is essential for the dissemination of evidence-based practices, yet therapist adherence and competence are seldom assessed. We examined the quality of delivery of cognitive-behavioural therapy (CBT) in routine psychiatric care for depression and anxiety disorders, considering therapist adherence and competence, and therapy effectiveness, as well as their associations.

**Methods:**

Twenty-nine therapists recruited 85 patients with a principal diagnosis of depression or anxiety disorder from two routine psychiatric outpatient clinics in Stockholm, Sweden. Therapist adherence was assessed mid-CBT by observers and post-CBT by patients and therapists, respectively, using an instrument developed as part of the present study. Therapist competence was assessed using role-plays with a standardised patient. Patients rated symptoms, functional impairment, and global health pre- and post-CBT. Linear mixed models were used to analyse associations.

**Results:**

Therapist adherence was high according to patients, moderate to high according to therapists, and moderate according to observers. Most therapists demonstrated competence in CBT, as assessed using the Cognitive Therapy Scale-Revised (*M* = 40.5, *SD* = 6.5; 76% passed the ≥ 36 points competence threshold). Patients improved significantly from pre- to post-CBT across outcome measures (Cohen’s *d*s = 0.80 – 1.36). Neither therapist adherence nor competence was associated with patient outcomes.

**Conclusions:**

In routine psychiatric care, therapists delivered CBT with adherence, competence, and improvements for patients with depression and anxiety disorders, on par with previous research results in controlled settings. The implications for quality assessment and improvement are discussed.

**Trial registration:**

ClinicalTrials.gov NCT03625024 10/08/2018.

**Supplementary Information:**

The online version contains supplementary material available at 10.1186/s12888-024-06328-4.

## Introduction

Depression and anxiety disorders are among the most common psychiatric disorders [1, 2], and psychiatric disorders pose greater burdens than any other disease [3]. Meanwhile, access to and quality of care are suboptimal [4]. Quality of care includes both the technical and interpersonal performance of healthcare providers [5], with the purpose of improving the health of patients.

According to Donabedian [5], assessment of quality of care should include multiple levels and different perspectives to provide a more accurate, faceted picture, which helps us to interpret findings. Thus, the quality of healthcare can be assessed on a structural level by examining the qualifications of staff, on a process level by reviewing the practice of healthcare, and on an outcome level by evaluating the improved health and functioning of patients [4, 6], as well as from the perspectives of patients, therapists, and observers. Despite the perceived importance of therapist adherence and competence in disseminating high-quality treatments, neither is commonly evaluated in psychiatric care [7].

To bridge the research-clinician gap, evidence-based treatments have been identified and incorporated into psychiatric healthcare guidelines for major depressive disorder (MDD) and anxiety disorders, including obsessive–compulsive disorder (OCD), posttraumatic stress disorder (PTSD), generalised anxiety disorder (GAD), panic disorder (PD), and social anxiety disorder (SAD). Cognitive-behavioural therapy (CBT) is a recommended treatment for these disorders in various countries [e.g., 8–11]. Their effectiveness has been demonstrated in routine psychiatric care, with moderate to large within-group effect sizes for depression and anxiety disorders [12, 13].

CBT is an umbrella term for therapies based on similar treatment principles. Thus, the guidelines for specific disorders may include CBT variations, for example, behavioural activation or cognitive therapy for MDD, exposure and response prevention for OCD, and cognitive processing therapy, cognitive therapy, or prolonged exposure therapy for PTSD [8]. With some variability, the different CBT protocols commonly include similar specific treatment techniques (e.g., exposure or cognitive restructuring) and procedures (e.g., agenda setting or home assignments).

Therapist adherence refers to the degree to which therapists deliver the techniques and procedures prescribed [14]. In a study of 317 CBT therapists, only 11% reported frequently using protocols in their work, while 30% reported never using them [15]. Similarly, community-based therapists reported using only some of the prescribed CBT interventions a few years after their CBT training [16]. The underuse of strategies such as exposure exercises or homework [16], is consistent with Waller’s [17, 18] observation that therapists tend to drift towards more talking and less active interventions aimed at behavioural change. Even in randomised controlled trials, non-supported modifications of treatments were common [19] and adherence decreased over CBT sessions [20]. However, therapist adherence should not be mistaken for an inflexible, rigid use of treatment protocols. Rather, patients may benefit from a degree of flexibility within treatment fidelity [21], where the therapist is guided by a predefined set of principles while applying a broad set of methods tailored to the individual case [22]. Hence, we believe therapist adherence to generic treatment principles can coexist with other desirable therapist behaviours, such as responsiveness and sound clinical judgment.

The European Psychiatric Association proposes that adherence to national guidelines be assessed using patient and therapist ratings of therapy content [7]. Similarly, triangulation of results from patient, staff, and observer perspectives has been proposed in a study that reported low agreement between patient and therapist ratings of content in inpatient psychiatric healthcare [23]. Patients have reported receiving both supported techniques and procedures, as well as those lacking empirical support [24, 25].

Although therapist adherence is often assumed to be associated with better patient outcomes, it has not been supported in meta-analyses [14, 26, 27]. Therapist adherence is typically assessed based on specific treatment protocols. However, most protocols share similar treatment principles, techniques, and procedures. Alternatively, assessing adherence to generic CBT techniques and procedures, on a continuous (rather than dichotomous) scale could be more useful. This approach would be more feasible in routine psychiatric care, where comorbidity is high, flexibility within adherence is common, and the use of a specific protocol is not always known. It also offers pragmatic advantages, as raters need only be trained in one tool since a generic measure can be applied across various disorders, increasing sample sizes.

While therapist adherence refers to whether a treatment is delivered as intended, therapist competence refers to the skill with which the prescribed methods are delivered [14]. Interestingly, to our knowledge, few studies have examined CBT competence among therapists in routine psychiatric practice. However, several studies have reported that therapist competence improves with training [28] and that a majority of therapists pass a competence threshold after training [29–31]. Studies have examined therapist competence as a predictor of patient outcomes. When including various psychotherapies, the competence–outcome association was either small (*r* = 0.17, *d* = 0.34) [27] or non-significant (*r*s = 0.03–0.07) in meta-analyses [14, 27]. However, a meta-analysis of CBT studies [26] found a moderate significant effect (*r* = 0.38) of competence–outcome for depression and a small significant effect (*r* = 0.24) for mixed disorders [26]. Most studies based their assessments on real patient sessions, potentially allowing patient- and session-related factors to influence therapist behaviour, thus requiring multiple sessions to rate therapist competence reliably [32].

Standardised role-plays have been recommended to reduce real patient variability [33] and were comparable to sessions with real patients in assessing therapist CBT adherence [34]. Role-plays with standardised patients have demonstrated feasibility, reliability, and validity in competence assessments within psychiatry [35], clinical psychology [36], and CBT for practicing therapists [37, 38]. They have successfully been used to show CBT competence development among practicing therapists during training [38–40]. Moreover, therapists found their role-play performance to resemble their clinical practice [38], while script adherence and character fidelity have been achieved through effective training of standardised patients [37].

Research examining whether therapist adherence and competence are associated with patient outcomes should control for therapeutic alliance as a confounding variable [14]. Yet, it has been controlled in a minority of studies [27]. A therapeutic alliance refers to the working relationship between the therapist and the patient, that is, their bond and agreement on therapy goals and tasks [41]. A meta-analysis of 72 CBT studies found a small, significant correlation of alliance–outcome across disorders (*r* = 0.20) [42]. Competence, alliance, and outcome seem to be associated, as the competence-outcome relationship was smaller in studies controlling for therapeutic alliance [14].

In conclusion, CBT is an evidence-based treatment for depression and anxiety disorders, often recommended as a first-hand choice, and it should matter whether CBT is delivered as intended, i.e., that therapists adhere to prescribed techniques and procedures, applying them with skill. However, research results have thus far been mixed.

The first aim of the present study was to assess the quality of CBT for depression and anxiety disorders in routine, psychiatric care in Stockholm, Sweden. At the structural level, we examined therapist CBT competence. At the process level, we examined therapist adherence to generic CBT techniques and procedures. At the outcome level, we examined the within-group effectiveness of CBT regarding symptom reduction, functional impairment, and quality of life. The second aim was to investigate associations between therapist competence, therapist adherence, and patient outcome.

The clinics included in this study emphasise the need for evidence-based psychiatric practice, using standardised psychiatric assessments, pre-specified treatment protocols, and web-based evaluations of patient outcomes. All the therapists were licensed clinical psychologists who had CBT training and formal qualifications to provide CBT for depression and anxiety disorders. Despite efforts to implement high-quality care, therapist adherence and competence have not yet been examined in these clinics. Our hypotheses were as follows: 1) therapists adhere to generic CBT techniques and procedures, 2) therapists are competent in delivering CBT, 3) CBT is delivered with within-group effect sizes of *d* > 0.5 across patient outcomes, and 4) therapist adherence to and competence in generic CBT are positively associated with patient outcome.

## Method

### Design and setting

A longitudinal, observational study was conducted during the years 2018–2020 within two outpatient clinics, providing publicly-funded specialised psychiatric care for depression and anxiety disorders in Stockholm, Sweden. To maintain clinical representativeness, we used consecutive sampling, patients were referred through the usual clinical routes, and therapists were instructed to provide treatment as usual. Assessments were either limited to or incorporated with the clinics’ standard assessment procedures. All procedures of the present study complied with the relevant local ethical guidelines, as well as the World Medical Association Declaration of Helsinki. Approval was granted by the regional ethical review board of Stockholm (2018/472–31/5 and 2018/1309–32). The study protocol was preregistered at ClinicalTrials.gov (NCT03625024 10/08/2018).

### Participants

Twenty-nine therapists providing individual CBTs were included. All had received university-level basic CBT training, equivalent to one year of full-time studies, and were licenced clinical psychologists, except for two resident psychologists. Among the therapists, 22 (75.9%) were female and the average age was 31.1 years (*SD* = 6.0, range 26–52). On average, they had 2.4 years (*SD* = 3.0, range 0–12) of clinical experience delivering CBT and had received CBT supervision for 2.0 years (*SD* = 2.2, range 0–10). The descriptive analysis of therapist competence levels is based on data from all therapists (*n* = 29).

Six therapists did not recruit any patients, due to promotion, parental leave, high workload, a lack of eligible patients, or their patients declined participation in the study. The remaining 23 therapists each recruited and treated a mean of 3.7 patients (*SD* = 2.9, range 1–11). Analyses including therapist adherence or patient outcomes only include data from therapists who recruited patients (*n* = 23). There were no statistically significant differences between recruiting and non-recruiting therapists regarding age, training, or competence levels.

Eighty-five consecutive patients were included. All eligible patients, referred to a participating therapist for CBT between August 2018 and February 2020, were informed of the study. All patients had undergone a comprehensive clinical and functional assessment, including semi-structured interviews [43, 44], patient-reported measures, and psychiatric history taking. Typically, the psychiatric assessment was conducted by a separate clinician and resulted in diagnoses and subsequent referrals for treatment. The psychiatric assessment was reviewed, and sometimes revised, by the treating therapist before commencing treatment. The inclusion criteria were a principal diagnosis of MDD (*n* = 12, 14.1%), OCD (*n* = 25, 29.4%), PTSD (*n* = 9, 10.6%), GAD (*n* = 23, 27.1%), PD (*n* = 11, 12.9%), or SAD (*n* = 5, 5.9%) according to the Diagnostic and Statistical Manual of Mental Disorders, Fifth Edition (American Psychiatric Association, 2013), referred to individual face-to-face CBT. Any psychotropic medication was encouraged to remain stable two months before CBT started and until post-assessments. An additional seven patients provided informed consent but either did not meet the inclusion criteria (*n* = 5) or met the exclusion criteria, which were a) acute suicidal ideation (*n* = 1), b) current substance use disorder (*n* = 1), and c) concurrent psychological treatment (*n* = 0). The patient characteristics are shown in Table 1. Among the 130 patients informed about the study, 38 (29.2%) declined to participate. Reasons included discomfort with session recording (*n* = 18, 47.4%, with 4 citing social anxiety and 2 obsessive, magical thinking), fear of being identified (*n* = 3, 7.9%), unwilling to complete additional self-reports (*n* = 3, 7.9%), being too tired (*n* = 2, 5.3%), or no reason was provided (*n* = 10, 26.3%). Therapist characteristics (e.g., age, experience, or competence level) were not associated with the number of patients they invited or the proportion of patients who accepted.

**Table 1:** Patient characteristics

	n	%
Age, years; *M* (*SD*)	35.2 (11.1)	
Symptom duration, years; *M* (*SD*)	12.0 (9.6)	
Number of psychiatric disorders; *M* (*SD*)	2.9 (1.1)	
Female	67	78.8
Born in Sweden	72	84.7
Married or had a live-in partner	38	44.7
Highest educational level		
9-year primary school	13	15.3
3-year secondary school	43	50.6
University degree	29	34.1
Employment		
Employed	45	52.9
On sick leave	20	23.5
Student	9	10.6
Unemployed	8	9.4
Retired	2	2.4
Parental-leave	1	1.2
Comorbid psychiatric conditions		
Major depression	51	60.0
Anxiety disorder	43	50.5
Autism spectrum disorder	8	9.4
Attention-deficit/hyperactivity disorder	17	20.0
No psychiatric comorbidity	7	8.2
Psychotropic treatment a month before and during CBT		
Stable dosage	43	50.6
Changed dosage or discontinued	14	16.5
New medication prescribed	11	12.9
No medication	16	18.8
Psychotropic medication		
Antidepressants	54	63.5
Anxiolytics	43	50.6
Benzodiazepines	27	31.8
Antipsychotics	13	15.3
Stimulants	10	11.8
Antiepileptics	7	8.2
Previously received CBT	45	52.9

*CBT* Cognitive-behavioural therapy

### Assessment

All measures were well-established and had previously been validated with good psychometric properties [45–56], except for the recently developed adherence measure. Information on principal and comorbid diagnoses, number of CBT sessions, concurrent psychotropic medication, and treatment protocols were retrieved from patients’ medical records.

#### Development of the therapist adherence to cognitive-behavioural therapy scale

The Therapist Adherence to Cognitive-Behavioural Therapy scale (TACBT) was developed within the present study to assess therapist adherence to generic CBT techniques and procedures. We aimed to create a measure of non-protocol-specific CBT for various diagnoses, assessing a broad range of techniques and procedures, and rated on an ordinal scale (rather than a dichotomous adherent/non-adherent). During development, we reviewed multiple CBT protocols for depression and anxiety disorders commonly used in Swedish psychiatric healthcare [57–63], identified common techniques and procedures, and synthesised them into different items. We also reviewed the research on therapist adherence to CBT, drawing inspiration from established instruments. These included the Multitheoretical List of Therapeutic Interventions [64], which assesses psychotherapy content across different orientations; the Collaborative Study Psychotherapy Rating Scale [65], which measures adherence to CBT for depression; and the Cognitive-Behavioral Therapy Adherence Scale [66], which evaluates adherence to specific CBT-protocols. During the developmental process, the TACBT was reviewed by a panel of CBT experts and several non-professionals, resulting in minor changes to item wording.

The TACBT assesses the use of CBT techniques and procedures using non-professional language and, for some items, professional terms in brackets. The 26 items are rated on a 5-point Likert scale (0 = *not at all*, 1 = *to a small degree,* 2 = *to a moderate degree*, 3 = *to a high degree*, and 4 = *to a very high degree*). The TACBT has four subscales: structure (9 items covering agenda setting, agenda adherence, homework setting, homework follow-up, in-session summarising, session evaluation, therapy goal setting, therapy goal evaluation, and maintenance planning), conceptualisation (6 items covering rationale, therapeutic model, antecedents/triggers, origins and maintaining factors, functional analysis, and the patient’s view of their problems), behavioural techniques (4 items covering planning of exposure or behavioural activation, in-session exposure, skills training, and behavioural rehearsal), and cognitive techniques (7 items covering interpretation of events, cognitive errors, identifying of beliefs, challenging beliefs, behavioural experiments, alternative beliefs, and problem-solving).

We created patient, therapist, and observer versions of each item (i.e., TACBT-p, TACBT-t, and TACBT-o; see Supplementary material), allowing multiple perspectives on therapist adherence. In the present study, both patient and therapist versions were used to evaluate the full course of treatment following its completion. Therefore, the patient and therapist versions are almost identical. Subscale and total sum scores were calculated, providing a range for TACBT-p and TACBT-t total scores of 0–104.

In the present study, the observer version was used to evaluate a single session. The fifth treatment session was audio-recorded and therapists were informed that it would be assessed for “quality”, an intentionally vague description on our part, without any further information or instructions, as to minimise influence on therapist behaviours. All recorded sessions were rated within a short timeframe by trained CBT experts. Expert rating proved more efficient using a simplified item wording combined with a more detailed example of what would constitute a very high degree of adherence (i.e., a maximum score) for each item. The observer version also included an additional brief description of response options.

The raters were licensed psychologists and trainers in advanced-level CBT education who had participated in eight hours of training and calibration based on three CBT practice sessions. No patients with MDD were included in the calibration sessions, after which we found an inconsistency in the coding of behavioural activation (specifically, avoidance patterns and alternative coping). This ambiguity in the item of exposure and activation planning was clarified (see Supplementary material), and the item was recoded for all patients with a principal diagnosis of MDD (*n* = 12). Raters were only informed of the patient’s principal diagnosis and that it was their fifth CBT session. The distinction between therapist adherence and competence was thoroughly discussed and practiced during rater training to ensure they were separated in ratings.

For single-session ratings, a structural subscale sum score was calculated, since the structural items were deemed relevant for all sessions of a treatment. In a single session, however, time is limited and a therapist would focus on a few techniques or procedures from the conceptual, cognitive, or behavioural items, rather than incorporating all available techniques and procedures into every single session. Thus, the conceptual, behavioural, and cognitive subscales were calculated based on the sum of the two highest item ratings of each subscale. To evaluate adherence from different perspectives (i.e., patient, therapist, and observer), item means were calculated for subscales and total scores. Four items (i.e., goal setting, goal evaluation, maintenance planning, and patient perspective on their problems) were deemed irrelevant for the single session and were thus removed from the TACBT-o, providing a total score range of 0–48.

The internal consistency was excellent across all items of the TACBT-p (α = 0.95; across subscales α = 0.73–0.93), good for the TACBT-t (α = 0.84; across subscales α = 0.61–0.82), and acceptable for the TACBT-o structural subscale (α = 0.72; not relevant for other TACBT-o scores since they were based on the top two item ratings in each subscale; thus, the items included in those scores differed between patients). The TACBT-o demonstrated good interrater reliability (intraclass correlation coefficient [ICC] = 0.75–0.79 across four raters; two-way random effects model, absolute agreement, single measurement).

#### Assessment of therapist competence

We employed a standardised patient approach, to create uniform conditions for therapists to demonstrate competence. Therapist competence has been found to vary between patients and over sessions in actual clinical settings [14], why standardised role-plays are a feasible method to minimise patient and session variability [32, 33, 67]. Therapists conducted a CBT role-play session with a standardised patient, that is, an actor portraying a patient with a specific set of presenting problems indicative of panic disorder with comorbid depression. The standardised patient suffered frequent panic attacks with severe anxiety and was increasingly isolated. Therapists were provided with written background information on the patient, the treatment up to this point, and homework for the session. They conducted a complete 45-min session of CBT, designed to constitute their fourth treatment session. The role-play was conducted in a therapy room at the clinic of the participating therapist, to ensure a familiar setting. The actors were four psychology students in their second to fourth year, who had received three hours of training. They followed detailed written instructions, including scripted responses to potential therapist behaviours. To ensure the stability of actors’ behaviours, they were to reread the manuscript before each role-play and were regularly monitored by the first author. The video-recorded CBT role-play was assessed for CBT competence by a separate group of observers, distinct from the previously mentioned adherence raters.

Therapist competence in CBT was assessed with the Cognitive Therapy Scale-Revised [CTS-R; [45]. The 12 items are rated on a scale from 0 (incompetent) to 6 (expert), ranging 0–72 with a competence threshold of ≥ 36. In the present study, the interrater reliability was good to excellent (ICC = 0.85–0.95 for two raters across three practice role-plays; two-way random effects model, absolute agreement, single measurement). The raters were psychotherapists, supervisors, and trainers in advanced-level CBT education. They participated in a two-day CTS-R workshop, followed by repeated training, calibration, and monitoring. Training included thorough discussion and practice in distinguishing therapist competence from adherence, to ensure separation. Raters were independent of the study, received no information on therapists, and were distinct from the psychologists who rated therapist adherence.

#### Patient outcome measures

Depressive symptoms were assessed in all patients with the Patient Health Questionnaire-9 [PHQ-9; [46]. The PHQ-9 score was also used as a diagnosis-specific outcome for patients with a principal MDD diagnosis. Nine items are rated on a scale from 0 (not at all) to 3 (nearly every day), ranging 0–27 with a recommended clinical cut-off of ≥ 10 [46]. The internal consistency at pre-assessment was good (α = 0.80) in the current study.

Obsessions and compulsions were assessed in patients with a principal OCD diagnosis, using the Obsessive Compulsive Inventory Revised [OCI-R; [47]. The 18 items are rated on a scale from 0 (not at all) to 4 (extremely), ranging 0–72 with a recommended clinical cut-off of ≥ 21 [47]. The internal consistency at pre-assessment was good (α = 0.89).

Posttraumatic stress symptoms were assessed in patients with a principal PTSD diagnosis using the Posttraumatic Stress Disorder Checklist for DSM-5 [PCL-5; [48]. The 20 items are rated on a scale from 1 (not at all) to 5 (extremely), ranging 20–100, with a recommended cut-off of ≥ 33 [68, 69]. The internal consistency at pre-assessment was excellent (α = 0.95).

Worry symptoms were assessed in patients with a principal GAD diagnosis using the Penn State Worry Questionnaire [PSWQ; [70]. The 16 items are rated on a scale from 1 (not at all typical) to 5 (very typical of me), ranging 16 − 80 with a recommended cut-off of ≥ 45 [49]. The internal consistency at pre-assessment was acceptable (α = 0.67).

General anxiety symptoms were assessed in all patients with the Generalized Anxiety Disorder Assessment 7 [GAD-7; [50]. The GAD-7 score was also used as a diagnosis-specific outcome for nine (39.1%) patients with a principal GAD diagnosis who did not complete their PSWQ assessments. Seven items are rated on a scale from 0 (not at all) to 3 (nearly every day), ranging 0–21, with a recommended clinical cut-off of ≥ 8 for GAD and anxiety disorders [71, 72]. The internal consistency at pre-assessment was good (α = 0.80).

Panic symptoms and related avoidance were assessed in patients with a principal PD diagnosis using the Panic Disorder Severity Scale Self-Rated [PDSS-SR; [51]. Seven items are rated on a scale from 0 (not at all) to 4 (extreme), ranging 0–28, with a recommended cut-off of ≥ 8 [52]. The internal consistency at pre-assessment was good (α = 0.83).

Social anxiety symptoms and avoidance were assessed in patients with a principal SAD diagnosis, using the Liebowitz Social Anxiety Scale Self-Report [LSAS-SR; [73]. Levels of fear/anxiety from 0 (none) to 3 (severe) and avoidance from 0 (never) to 3 (usually) are rated for 24 social situations, providing a range of 0 − 144, with a recommended cut-off of ≥ 60 [53]. The internal consistency at pre-assessment was poor (α = 0.45).

Functional impairment was assessed using the 12-item WHO Disability Assessment Schedule 2.0 [WHODAS 2.0; [74], which covers the domains of cognition, mobility, self-care, getting along, life activities, and participation. Items are rated on a scale from 0 (none) to 4 (extreme or cannot do), providing a range of 0–48 using the simple scoring method. The internal consistency at pre-assessment was good (α = 0.87).

Global health was assessed using the EQ-5D visual analogue scale [EQ-VAS; [75], ranging from 0 (the worst health you can imagine) to 100 points (the best health you can imagine).

#### Assessment of therapeutic alliance

Therapeutic alliance was assessed with the Working Alliance Inventory Short-form Revised [WAI-SR; [56]. The 12 items are rated on a scale from 1 (seldom) to 5 (always), ranging 12–84. The internal consistency in the present study was excellent (α = 0.94).

### Procedure

All therapists and patients eligible for participation received oral and written information about the study and provided written informed consent before participating. Before patient recruitment, therapists conducted a CBT role-play session with a standardised patient. Observers then assessed the recorded session for CBT competence.

Therapists informed all their eligible patients of the study and obtained written informed consent. Patients completed web-based self-report measures pre-CBT and post-CBT, including a diagnosis-specific symptom measure, depending on their principal diagnosis, as well as the PHQ-9, GAD-7, WHODAS, and EQ-VAS. Pre-assessments were conducted within 14 days before or after CBT onset, of which 63% were within a week and 40% within three days. Post-assessments were conducted within 30 days before or after the final CBT session, of which 72% were within a week and 56% within three days.

The fifth treatment session was audio-recorded and assessed for therapist adherence by an independent observer. During the same session, patients reported on the therapeutic alliance. At the end of CBT, both the patient and therapist rated the therapist’s adherence to CBT regarding complete treatment.

Eleven patients were still in treatment when the COVID-19 pandemic began in March 2020, with the last treatment completed by December 2020. As restrictions in Sweden were kept to a minimum, most treatments proceeded as planned. While some exercises were modified and session rescheduling increased, only one patient paused treatment. After reviewing each case, we concluded that all treatments had been completed. However, we cannot rule out that the adherence rating or patient outcomes were affected in these individual cases.

### Statistical analysis

The SPSS (version 26, SPSS Inc., Chicago, IL) was used for all the analyses. To examine agreement between patient and therapist ratings of therapist adherence to CBT we calculated an intraclass correlation coefficient (ICC; one-way random effects model, absolute agreement, single measurement). To examine the relationship between therapist competence and therapist characteristics (i.e., years of age, clinical experience, and supervision experience) we ran Spearman rank-order correlations.

Linear mixed models (LMMs) were used to estimate the effect of time on patient outcomes and investigate potential associations with therapist adherence and competence. We chose the LMM because it is recommended for nested data and for handling missing data appropriately [e.g., [76]. Thus, pre- and post-assessments of outcome variables were nested within patients within therapists and clinics. Because of the small number of patients per principal diagnosis, we calculated standardised *z*-scores for diagnosis-specific outcomes (i.e., PHQ-9, OCI-R, PCL-5, PSWQ or GAD-7, PDSS-SR, and LSAS-SR) and collapsed them into a composite score. In each analysis, we set the outcome measure as the dependent variable (i.e., diagnosis-specific composite score or the raw total score of the PHQ-9, GAD-7, EQ-VAS, or WHODAS). Thus, for eleven patients with principal MDD, we used their PHQ-9 total scores both as part of the composite score and as raw scores to measure the effect of CBT on depressive symptoms for all patients. Similarly, for the nine patients with principal GAD who did not complete their PSWQ assessments, we used the GAD-7 both as part of the composite score and as raw scores to measure the effect of CBT on general anxiety.

Only participants who completed their pre-assessment of an outcome measure were included in each analysis (i.e., 8.2% – 29.4% [*n* = 7 – 25] were excluded across analyses). Missing post-assessments (i.e., 3.8% – 13.3% [*n* = 3 – 8] of included patients across analyses) were estimated using maximum likelihood estimation. The maximum likelihood method was used to estimate the model parameters. The fit of each model to the observed data was evaluated using the likelihood ratio test, with significance set at 0.05. A model with a significantly better fit than the previous model was retained. We started with a basic model including a fixed intercept. We successively added a random intercept, a fixed slope (i.e. a main effect of time), and a random slope. Finally, we added a time-by-variable interaction term, to examine variables associated with patient outcomes, which was removed from the model before adding a new variable. The variables were: symptom duration, number of psychiatric diagnoses, therapeutic alliance (WAI-SR total scores), therapist, therapist experience, therapist adherence (TACBT-p, TACBT-t, and TACBT-o total scores), therapist competence (CTS-R total scores), and clinic. In the case of significant interaction effects for competence or adherence, the plan was to control for the influence of therapeutic alliance (WAI-SR total score) by adding it to the model, as recommended by Webb, DeRubeis, and Barber [14]. The standardised effect size for within-group effects at post-assessment was calculated as Cohen’s *d* for LMM based on the formula recommended by Feingold [77]; Eq. 1], using the unstandardized coefficient for the slope, thus adjusting for the within-group correlation of pre- and post-assessment values, and the pre-assessment sample standard deviation. For model-based *d*, 95% confidence intervals (CIs) were calculated using the formulas provided in Feingold [77]; Eqs. 7 and 8]. We assessed the between-therapist variance, based on an LMM with a random intercept and a fixed slope. To compute the ICC, we divided the between-therapist (random intercept) variance by the total variance (sum of between-therapist variance and within-therapist variance [the residuals]), as recommended by Tabachnick and Fidell [78]; Eq. 14].

Patient treatment response was assessed using the reliable change index [RCI; [79], calculated using the sample standard deviation at pre-assessment and the internal consistency of each outcome measure (see Assessments). An RCI of *z* ≤ -1.96 indicates reliable improvement, while an RCI of *z* ≥ 1.96 indicates reliable deterioration. Recovery, or clinical significance, was defined as a statistically reliable improvement, combined with moving from above the clinical cut-off before treatment to below the cut-off after treatment.

## Results

### Cognitive-behavioural therapy in practice

Overall, patients received a median of 18.5 sessions of CBT (interquartile range [IQR] 13.0–24.0). In 81 (95.3%) of the patients’ records, therapists reported having used specific CBT protocols in accordance with guidelines: behavioural activation for MDD [57], exposure and response prevention for OCD [58], prolonged exposure for PTSD [59], CBT for GAD [60], cognitive therapy [61] or CBT [62] for PD, and CBT for SAD [63]. However, in one PD treatment and three SAD treatments, the therapist neither specified a protocol nor described the therapy content sufficiently to conclude whether a specific treatment was delivered. Moreover, in conjunction with treatment for their principal diagnosis, 17 patients (20.0%) received additional treatment targeting a comorbid diagnosis. Specifically, 15 patients with comorbid MDD received elements of behavioural activation, and two patients with comorbid GAD received elements of CBT for GAD. Patients who received additional treatments received a median of 21.0 sessions (IQR 17.5–26.5), which was non-significantly greater than the 17.5 sessions for single treatments (IQR 12.8–22.5; Mann–Whitney U = 688.00, *p* = 0.061).

### Therapist adherence

Therapist adherence to CBT was high according to patients, and moderate to high according to therapists. Meanwhile, observers of a single CBT session found therapist adherence moderate (Table 2). On average, structural and conceptual items received higher scores than behavioural and cognitive items. The agreement between patient and therapist ratings of complete treatments was poor (ICC = -0.14, 95% CI [-0.35, 0.10]).

**Table 2:** Therapist adherence to cognitive-behavioural therapy from different perspectives

	Complete therapy		Single session
	Patient (*n* = 76)	Therapist (*n* = 77)	Observer (*n* = 81)
	*M* (*SD*)	*M* (*SD*)	*M* (*SD*)
Total score	87.72 (16.25)	70.16 (11.19)	25.57 (7.79)^a^
Total item mean	3.37 (0.62)	2.70 (0.43)	2.13 (0.65)^a^
Structural subscale	31.75 (5.30)	26.18 (5.22)	13.09 (4.59)
Subscale item mean	3.53 (0.59)	2.91 (0.58)	2.18 (0.77)
1. Agenda setting	3.47 (0.90)	2.97 (1.01)	2.57 (1.52)
2. Agenda adherence	3.50 (0.70)	2.84 (0.75)	2.79 (1.58)
3. Homework setting	3.88 (0.36)	3.68 (0.52)	3.43 (0.89)
4. Homework follow-up	3.76 (0.51)	3.44 (0.70)	3.10 (1.04)
22. In-session summarising	3.32 (1.01)	1.91 (1.04)	0.83 (1.06)
23. Session evaluation	3.07 (1.19)	1.51 (1.18)	0.37 (0.83)
24. Therapy goal setting	3.80 (0.46)	3.55 (0.72)	n/a
25. Therapy goal evaluation	3.57 (0.82)	3.18 (1.02)	n/a
26. Maintenance planning	3.38 (1.02)	3.10 (1.30)	n/a
Conceptual subscale	21.45 (3.65)	18.87 (2.89)	4.93 (2.26)^a^
Subscale item mean	3.57 (0.61)	3.15 (0.48)	2.46 (1.13)^a^
5. Rationale	3.71 (0.61)	3.52 (0.58)	2.26 (1.40)
6. Therapeutic model	3.57 (0.77)	3.39 (0.69)	1.65 (1.33)
7. Antecedents/triggers	3.61 (0.77)	2.87 (0.85)	1.81 (1.47)
8. Origins and maintaining factors	3.49 (0.92)	3.21 (0.68)	0.37 (0.80)
9. Functional analysis	3.36 (0.86)	2.96 (0.85)	1.56 (1.36)
10. Patient view of problems	3.72 (0.58)	2.92 (0.74)	n/a
Behavioural subscale	12.15 (3.36)	9.60 (2.92)	4.73 (2.77)^a^
Subscale item mean	3.04 (0.84)	2.40 (0.73)	2.36 (1.39)^a^
11. Exposure/activation planning	3.83 (0.44)	3.62 (0.56)	2.86 (1.44)
12. In-session exposure	3.11 (1.17)	2.36 (1.35)	1.53 (1.84)
13. Skills training	3.21 (1.01)	2.42 (1.08)	0.40 (0.92)
14. Behaviour rehearsal	2.01 (1.58)	1.19 (1.16)	0.00 (0.00)
Cognitive subscale	22.37 (6.03)	15.51 (5.18)	2.83 (2.15)^a^
Subscale item mean	3.20 (0.86)	2.22 (0.74)	1.41 (1.07)^a^
15. Interpretation of events	3.13 (1.05)	2.40 (0.80)	0.73 (1.06)
16. Cognitive errors	3.16 (1.13)	1.73 (1.14)	0.30 (0.81)
17. Identifying beliefs	3.14 (1.06)	1.99 (1.24)	1.06 (1.19)
18. Challenging beliefs	3.42 (0.91)	2.31 (1.13)	0.25 (0.64)
19. Behavioural experiments	2.97 (1.10)	2.38 (1.12)	0.28 (0.79)
20. Alternative beliefs	3.20 (1.02)	2.27 (0.96)	0.69 (1.09)
21. Problem-solving	3.33 (0.92)	2.43 (1.07)	0.28 (0.68)

^a^Based on the two highest item scores in the conceptual, behavioural, and cognitive subscales

### Therapist competence

Therapists demonstrated a mean competence score on the CTS-R of 40.5 (*SD* = 6.5, range 28–54) in standardised role-plays. The competence threshold (i.e., a CTS-R total score ≥ 36 points) was passed by 75.9% (*n* = 22). Therapists’ competence level was not significantly correlated with years of CBT experience (*r* = -0.33, *p* = 0.077), years of CBT supervision received (*r* = -0.33, *p* = 0.080), or age (*r* = -0.12, *p* = 0.522).

### Patient outcomes

To estimate the effect of time on patient outcomes, we performed separate LMMs for each dependent variable nested within patients within therapists. The model that provided the best fit included a random intercept and a fixed slope, but no random slope. The results indicate significant improvements over time across outcomes (*d*s = 0.80–1.30), see Table 3. Neither patient symptom duration, number of psychiatric diagnoses, therapist, therapist CBT experience, therapeutic alliance, nor psychiatric clinic had any significant effects on outcomes (i.e., the time-by-variable interaction term was non-significant).

**Table 3:** Patient-reported outcomes from pre- to post-assessments

	Pre-assessment		Post-assessment		Linear mixed model		Effect size
	n	M (SD)	n	M (SD)	df	F	*d* [95% CI]
Diagnosis-specific measures depending on the principal diagnosis							
PHQ-9	11	18.82 (3.98)	9	9.50 (5.16)			
OCI-R	24	31.77 (16.05)	24	22.79 (12.96)			
PCL-5	6	36.83 (16.15)	6	19.83 (21.08)			
PSWQ	11	71.73 (5.71)	11	55.32 (13.16)			
GAD-7^a^	9	11.67 (4.09)	9	5.22 (2.40)			
PDSS-SR	11	15.86 (5.23)	11	4.95 (2.83)			
LSAS-SR	5	101.60 (8.65)	4	64.75 (27.49)			
Composite score (*z*)^b^	77	0.60 (0.84)	74	-0.55 (0.86)	76.84	96.83^*^	1.36 [1.64, 1.09]
General measures for all patients							
PHQ-9	70	14.58 (5.22)	64	8.80 (5.66)	68.23	63.07^*^	1.10 [1.38, 0.82]
GAD-7	75	13.20 (4.66)	68	7.11 (4.94)	74.38	89.45^*^	1.30 [1.57, 1.03]
WHODAS 2.0	60	18.97 (8.64)	52	11.37 (9.18)	57.91	34.17^*^	0.87 [1.16, 0.57]
EQ-VAS	71	45.54 (21.24)	67	62.19 (20.03)	69.39	50.65^*^	0.80 [0.57, 1.02]

*PHQ-9* Patient Health Questionnaire-9, *OCI-R* Obsessive Compulsive Inventory-Revised, *PCL-5* Posttraumatic Stress Disorder Checklist for DSM-5, *PSWQ* Penn State Worry Questionnaire, *GAD-7* Generalized Anxiety Disorder Assessment 7, *PDSS-SR* Panic Disorder Severity Scale Self-Rated, *LSAS-SR* Liebowitz Social Anxiety Scale Self-Report, *WHODAS 2.0* WHO Disability Assessment Schedule 2.0, *EQ-VAS* EQ-5D visual analogue scale

^a^For patients with a principal generalised anxiety disorder who did not complete their PSWQ assessments, we used the GAD-7 score as their diagnosis-specific outcome measure (*n* = 9/23)

^b^Because of the small number of patients per diagnosis, we calculated z-scores for diagnosis-specific outcomes, which were collapsed into a composite score and used for the linear mixed model

^*^p < .001

From pre- to post-assessment, 54.1% (*n* = 46) of patients reliably improved on their diagnosis-specific measure, including 31.8% who reliably recovered (*n* = 27). Meanwhile, 30.6% (*n* = 26) remained unchanged, 2.4% reliably deteriorated (*n* = 2), and data were incomplete for 12.9% (*n* = 11). Across all symptom measures combined (i.e., diagnosis-specific, PHQ-9, and GAD-7), 67.1% reliably improved (*n* = 57), including the 50.6% who reliably recovered (*n* = 43), on at least one of the measures, without reliably deteriorating on any of them. Meanwhile, 4.7% reliably deteriorated (*n* = 4) on one or more of the symptom measures, 16.5% remained unchanged (*n* = 14) across all symptom measures, and 11.8% (*n* = 10) had incomplete data for all symptom measures.

### Associations between therapist adherence/competence and patient outcomes

To examine the associations of therapist adherence and competence with patient outcomes, ratings of adherence and competence were added separately to the previously mentioned LMM with a random intercept and fixed slope. Patients were nested within therapists within clinics. Therapist adherence from each perspective (i.e., total scores on the TACBT-p, TACBT-t, and TACBT-o, separately) significantly improved model fit. However, the two significant interaction effects were both negligible: TACBT-t by time on z-scores of diagnosis-specific composite score, *F*(1, 69.33) = 4.77, *p* = 0.032, *d* = 0.03, 95% CI [0.00, 0.05] and TACBT-p by time on the WHODAS, *F*(1, 55.44) = 9.08, *p* = 0.004, *d* = 0.03 [0.01, 0.05]. Adding therapist competence (i.e., total scores on the CTS-R) to the previously mentioned model with a random intercept and fixed slope did not improve the model fit. These results indicate that therapist adherence or competence were not associated with patient outcomes. Thus, there was no reason to control for therapeutic alliance (*M* = 75.20, *SD* = 9.45, range 45–84) in the analyses by adding this variable to the models.

Between-therapist differences were associated to 1–19% of the variability in patient-reports, i.e., total scores of diagnosis-specific outcomes (ICC = 0.01, 95% CI [0.00, 0.99]), PHQ-9 (ICC = 0.17 [0.07, 0.36]), GAD-7 (ICC = 0.08 [0.02, 0.27]), WHODAS (ICC = 0.09 [0.02, 0.36]), EQ-VAS (ICC = 0.19 [0.08, 0.41]), WAI-SR (ICC = 0.09 [0.02, 0.37], and TACBT-p (ICC = 0.09 [0.01, 0.48]). Meanwhile, 39% of the variability in total scores of the TACBT-o (ICC = 0.39 [0.28, 0.51]) and 66% of the TACBT-t (ICC = 0.66 [0.58, 0.74]) was associated with between-therapist differences. This indicates, not surprisingly, a larger therapist effect when therapists rated their adherence than when patients rated therapist adherence, patient outcomes, or therapeutic alliance. Reversely, it indicates more within-therapist variability in patient-reports. However, the large confidence intervals demonstrate the need to interpret these findings cautiously. ICC could not be calculated for the CTS-R because competence was assessed once per therapist providing no within-therapist variability.

## Discussion

We aimed to assess the quality of CBT in routine psychiatric care for adults with depression and anxiety disorders. The study was based on the framework of quality assessment by Donabedian [5, 6]. As hypothesised, at the structural level, we found that most therapists were competent in delivering CBT as assessed during a standardised role-play. At the process level, we found that therapists adhered to CBT guidelines. Specifically, we found a moderate to high degree of adherence to generic CBT from patient, therapist, and observer perspectives. Also, therapists reported using recommended evidence-based treatment protocols in patient medical records. At the outcome level, we found that patient-reported symptoms, functional impairment, and global health improved from pre- to post-assessments (within-group *d*s = 0.80 – 1.36). However, contrary to our hypothesis, we found no significant associations between therapist adherence or competence and patient outcome.

Previous research suggests that therapist adherence is often low, with insufficient use of treatment protocols in clinical practice [15] and non-supported treatment modifications even in controlled settings [16, 19]. In contrast, our results indicate that in routine psychiatric care, with patients demonstrating high levels of comorbidity, therapist adherence can be achieved. A combination of organisational, therapist, and patient factors likely contributed to this. While standardised evidence-based care has gained importance over the last two decades in psychiatric services in Stockholm, the clinics in our study may not be fully representative across services. These two clinics have made notable long-term efforts to implement evidence-based care, in terms of prescribing diagnosis-specific treatment protocols, routinely evaluating patient outcomes, and engaging in a continued dialogue on the quality of care among therapists. All the therapists were CBT-trained clinical psychologists, opting for an employer with high regard for evidence-based and standardised practice. Although many services have undertaken comparable initiatives, the extent and impact differ within Stockholm, throughout Sweden, and internationally. Consequently, we cannot be certain about the generalisability of our results across clinical settings.

The patients in the present study were referred for treatment through routine clinical pathways after having undergone standardised semistructured assessments. They demonstrated high levels of comorbidity; 91.8% had multiple psychiatric diagnoses and almost one-third had a comorbid neuropsychiatric condition. Thus, they can be considered clinically representative of patients in specialised psychiatric care. Hence, it is not surprising that the number of sessions (*Mdn* = 18.5 sessions, IQR 13.0–24.0) was greater than advised in some of the treatment protocols reported, in which the suggested maximum of sessions is 15 to 24 [57–63]. Although the extension of treatment may indicate inefficient use of time, in this sample, it seems more likely to be a deliberate adjustment based on the patient’s needs, abilities, and comorbidity.

Interestingly, patients rated therapist adherence higher than their therapists, in contrast with previous findings where therapists rated content higher than their patients [23]. Systematic differences in staff and patient perspectives have been suggested, but not specified or explored [23]. There may be a systematic reporting bias, such as patients overestimating CBT content due to social desirability bias (even if they were informed that therapists would not see their ratings) or therapists underestimating it due to modesty or self-criticism, in line with findings of therapists tending to underestimate their skills [31, 80]. Patients could have lower expectations of the prevalence of some CBT techniques and procedures and provide higher ratings than therapists with more knowledge of treatment content. As an example, in-session summarising and session evaluation were reported to be high by patients, but less than moderate by therapists, and less than a little by observers. Perhaps, patients may not expect this to be more frequent in CBT than in other social interactions, while therapists and observers expect it to be done repeatedly, in dialogue, in all sessions. However, therapists reported a moderate level of behavioural and cognitive techniques throughout the treatment, revealing room for improvement.

Most therapists passed the competence threshold but a few therapists did not. This could be a cause for concern, implying cases of substandard quality. However, it was not associated with patient outcomes, and even lower CTS-R scores corresponded to those of an advanced beginner [i.e., CTS-R item mean > 2; [45]. Moreover, these competence levels are comparable to previous research on clinical therapists after training [29].

The results of this naturalistic study indicate improvements over time on par with previous efficacy randomized controlled trials [e.g., [81, 82] and nonrandomized effectiveness studies in similar settings with a comparable mean number of CBT sessions [i.e., 21.7–23.7 sessions (SDs = 9.9–11.9), compared to 20.6 sessions (SD = 10.3) in the present study; [12, 13]. Additionally, response rates are comparable to those observed in other naturalistic studies [83, 84]. Thus, the present uncontrolled study benchmarks the outcome of CBT in routine psychiatric care, for clinically representative patients with depression and anxiety disorders in Sweden against previous efficacy and effectiveness studies.

In the present study, CBT seems to have targeted not only diagnosis-specific symptoms but had a broad effect on anxiety and depressive symptoms. Typically, patients experienced reliable diagnosis-specific improvement (54%) and recovery (32%), which often coincided with reduced comorbidity. Yet, some patients demonstrated improvement (13%) and recovery (19%) only in comorbid anxiety or depressive symptoms. These results suggest that CBT targeting a principal diagnosis, may in some cases have a more pronounced clinical effect on comorbidity than on diagnosis-specific symptoms.

It seems plausible that delivering treatments as intended and with skill should affect patient outcomes, but if such an association exists, we were unable to detect it. We examined general CBT therapist adherence and competence, but perhaps these constructs should be operationalised based on the specific CBT protocol of each treatment, which a few studies indicate [85, 86]. Another possible explanation may be the complexity of the relationship. Branson et al. [87, 88] found that more patients improved and fewer patients deteriorated among the most competent therapists, with the reverse results among the least competent therapists. Thus, the importance of therapist adherence and competence may be negligible within a wider range of “good enough”. While linear correlations are usually assumed, McCarthy, Keefe, and Barber [89] found a curvilinear relationship of therapist adherence in depression; specifically, moderate levels of adherence improved outcomes. However, visual inspection did not indicate any such associations in our data.

While therapist adherence and competence are essential to treatment fidelity, results indicate they are not associated with therapy effectiveness. From a quality assurance perspective, it is reasonable to strive for high adherence and competence, as a way of implementing clinical guidelines and delivering effective treatments as intended and with skill. However, our results do not support that efforts to increase adherence and competence would affect patient outcomes. Other factors have been found to influence patient outcomes, such as initial symptom severity and comorbidity [84], waiting time and missed appointments [90], treatment dosage [13, 90], patient expectancies [91], and therapeutic alliance [42]. Our limited understanding of why some patients improve more than others underscores the ongoing need for research into factors influencing treatment effectiveness, a topic that has been a subject of inquiry in psychotherapy research for decades [92].

### Limitations of the study

The present study included a relatively small number of therapists with varied eligible numbers of patients. Consequently, the number of therapists (*n* = 23) and the patient-to-therapist ratio (*M* = 3.7, *SD* = 2.9, range 1–11) were inadequate to reliably estimate therapist effects on outcomes, if they exist [93]. Linear mixed modeling of small samples increases the risk of both Type I and Type II errors. The study was underpowered to reliably detect (or reject) potential associations at the patient, therapist, and clinic levels. Hence, it is important to interpret our results regarding these associations with caution.

As therapist competence tends to vary between patients and sessions [14], standardised patients can provide equal opportunities for therapists to demonstrate their range of skills [32]. The transferability of CBT competence between standardised and real patients is uncertain, with only two studies conducted indicating a weak association between the assessment methods in 88 and 14 cases, respectively [94, 95]. Meanwhile, therapists have found their role-play performance to resemble their clinical practice [38]. If role-plays lack generalisability to clinical practice, our assessment methods may have obscured a potential competence-outcome association. However, the use of standardised patients in psychotherapy and CBT is relatively recent, and further investigation is needed [94].

Despite instructions for therapists to assess outcomes at pre- and post-assessments, some assessments were completed earlier, later, or not at all. To assess the quality of routine clinical care, we minimised the impact of the research study by integrating assessments into routine practice and limited research-specific monitoring. Unfortunately, this may have promoted a less strict, “assessment-as-usual” approach to data collection among therapists, increasing variation in assessment timing and missing data. Thus, the results of the present study may have been affected by the exclusion of patients without pre-assessments, the estimation of missed post-assessments (3.8% – 13.3%), and generously allowing assessments to vary ± 14 days of CBT onset and ± 30 days of the last session, even if most assessments were conducted within a week. Pre-assessments were delayed for some patients and may have been affected by a potential early treatment response, as there is some evidence that so-called "sudden gains" predict post-treatment improvements [96]. However, patients improved across outcome measures, regardless of the number of excluded patients and estimated post-assessments. Any early treatment response would most likely deflate effect sizes, indicating that these limitations may not have affected the results, at least not considerably. Also, our outcome measures were limited to patient-report, as part of our efforts to assess the quality of “treatment as usual” and minimise our influence on routine practice. Still, a multimethods approach including clinician assessment of patient outcomes would have improved the study.

We used a measure of therapist adherence developed specifically for the study. The internal consistency was acceptable to excellent across the versions, and the interrater reliability for observers was good. Moreover, experts, clinicians, and non-professionals perceived it as comprehensible and to have good face validity. Thus, it demonstrated sound psychometric properties, but the subscales have not yet been validated in terms of factor structure, meaning that subscale scores and their interpretations rely on theoretical assumptions. As an example, the reported CBT protocols were mainly either behavioural [57–59] or cognitive [60, 61, 63], but we found no such distinction reflected in the respective subscale scores. This implies that therapists deliver CBT based on both cognitive and behavioural treatment principles rather than strictly adhering to the treatment protocol, or that there is an overlap in the TACBT formulation of cognitive and behavioural items.

Therapist adherence was assessed from multiple perspectives, as recommended [7, 23]. Therefore, we considered it suitable to use the same instrument for patients, therapists, and observers alike, thus enabling a triangulation of assessments. Unfortunately, in the present study, we were unable to use multiple sessions for observer ratings. While a single-session rating can provide an indication of therapist adherence, it will not cover all techniques and procedures of treatment and may not be representative. The single-session observer ratings should not be compared to the complete treatment scores of therapist and patient. To accommodate patient respondents, items used more concrete descriptions of therapist behaviour, such as “In sessions, we did things I find uncomfortable or usually avoid", which did not allow for a differentiation between “exposure in vivo” and “behavioural activation”. In particular, behavioural items proved difficult to differentiate in the patient version, why the TACBT includes more cognitive than behavioural items. Thus, the TACBT may benefit from further attempts to specify and add items on behavioural techniques.

Patients seem to have been able to complete the TACBT-p without difficulty, but their understanding of items and response choices should be examined. It may be difficult to assess the degree of a therapist activity, especially for patients new to therapy. To address this, we consulted non-professionals during development and made minor adjustments. We also considered a frequency-based scale but found it inadequate for most items, as it wouldn’t account for whether an activity was conducted to a suitable extent. However, further investigation is needed to ensure the TACBT’s clarity and ease of use for patients, along with a future validation of the TACBT and its subscales to investigate its psychometric properties in other settings and populations.

## Conclusions

This study found satisfactory levels of therapist adherence, therapist competence, and CBT effectiveness within routine psychiatric care for patients with depression and anxiety disorders. While potential areas for quality improvement would be increased use of behavioural and cognitive techniques, and competence development for a few of the therapists, this study did not provide any support that they will affect patient outcomes. Our naturalistic study benchmarks outcomes against efficacy [81, 82] and effectiveness [12, 13] studies. In conclusion, the present study demonstrated that high-quality CBT can be delivered within routine psychiatric care to clinically representative patients by competent therapists adhering to CBT principles to achieve the expected patient outcomes.

## Supplementary Information

Supplementary Material 1.

## Data Availability

The datasets generated and analysed during the current study are available from the last author upon reasonable request, subject to approval from of the participating clinics.

## Abbreviations

CBT: Cognitive-behavioural therapy
CI: Confidence interval
CTS-R: Cognitive Therapy Scale-Revised
DSM-5: Diagnostic and Statistical Manual of Mental Disorders, Fifth Edition
EQ-VAS: EQ-5D visual analogue scale
GAD: Generalised anxiety disorder
GAD-7: Generalized Anxiety Disorder Assessment 7
IQR: Interquartile range
LSAS-SR: Liebowitz Social Anxiety Scale Self-Report
MDD: Major depressive disorder
OCD: Obsessive-compulsive disorder
OCI-R: Obsessive Compulsive Inventory Revised
PCL-5: Posttraumatic Stress Disorder Checklist for DSM-5
PD: Panic disorder
PDSS-SR: Panic Disorder Severity Scale Self-Rated
PSWQ: Penn State Worry Questionnaire
PHQ-9: Patient Health Questionnaire-9
PTSD: Posttraumatic stress disorder
RCI: Reliable change index
SAD: Social anxiety disorder
TACBT: Therapy Adherence to Cognitive-Behavioural Therapy scale
TACBT-o: Therapy Adherence to Cognitive-Behavioural Therapy scale – observer version
TACBT-p: Therapy Adherence to Cognitive-Behavioural Therapy scale – patient version
TACBT-t: Therapy Adherence to Cognitive-Behavioural Therapy scale – therapist version
WAI-SR: Working Alliance Inventory Short-form Revised
WHODAS: WHO Disability Assessment Schedule
