# Supplementary material for: Quality of cognitive-behavioural therapy in routine psychiatric care: therapist adherence and competence, and patient outcomes for depression and anxiety disorders
Source: BMC Psychiatry. 2024 Dec 4;24:887. doi: 10.1186/s12888-024-06328-4 (PMC11616186; doi:10.1186/s12888-024-06328-4)
Supplement: Supplementary file 1 — Supplementary Material 1. [file 12888_2024_6328_MOESM1_ESM.pdf]

Centre for Psychiatry Research
Hillevi Bergvall & Benjamin Bohman 2018

Therapist Adherence to Cognitive-Behavioural Therapy – Patient version

The following statements describe activities which may have occurred in the therapy you have just received. Please rate how well each statement applies to the therapy using the scale below. Please choose only one response per item. There are no right or wrong answers.

	Not at all	To a small degree	To a moderate degree	To a high degree	To a very high degree
1. At the start of sessions, my therapist and I went over how the session should be spent (an agenda or session plan).	0	1	2	3	4
2. My therapist and I spent the session on what we had agreed upon at the start of the session.	0	1	2	3	4
3. My therapist and I agreed on homework between sessions (e.g., practicing behaviours or recording my thoughts, emotions, or events).	0	1	2	3	4
4. My therapist and I went over my homework since last session (e.g., exercises or recordings).	0	1	2	3	4
5. My therapist described why we focused on certain topics or exercises (i.e., provided a rationale).	0	1	2	3	4
6. My therapist explained how my thoughts, emotions, and behaviours influence each other.	0	1	2	3	4
7. My therapist and I explored triggers to my problematic thoughts, emotions, and behaviours.	0	1	2	3	4
8. My therapist and I explored the origins of my problems and what maintains them.	0	1	2	3	4
9. My therapist and I explored the consequences of my behaviours.	0	1	2	3	4
10. My therapist and I explored how I view my problems.	0	1	2	3	4
11. My therapist and I planned how I can change my behaviours between sessions (e.g., doing things I fear, being more active, meeting people, or reducing control).	0	1	2	3	4
12. In sessions I did things I find uncomfortable or avoid (e.g., exposing myself to objects, people, situations, or memories).	0	1	2	3	4
13. My therapist and I practiced new skills (e.g., being assertive, breathing training, or how to act in social situations).	0	1	2	3	4
14. My therapist and I role-played or rehearsed certain situations or behaviours together.	0	1	2	3	4
15. My therapist and I practiced finding alternative explanations to events or behaviours than those that first came to mind.	0	1	2	3	4
16. My therapist and I practiced noticing thinking errors or traps (e.g., black-and-white thinking, mind reading, or generalising).	0	1	2	3	4
17. My therapist and I explored how my problems are influenced by certain beliefs I have or rules I follow.	0	1	2	3	4
18. My therapist and I practiced challenging my thoughts or beliefs.	0	1	2	3	4
19. My therapist and I did exercises to test if my thoughts or beliefs were true.	0	1	2	3	4
20. My therapist and I practiced formulating new, more realistic thoughts or beliefs.	0	1	2	3	4
21. My therapist and I practiced solving problems.	0	1	2	3	4
22. My therapist and I repeatedly summarised our work during sessions.	0	1	2	3	4
23. My therapist and I evaluated the session at the end of sessions (e.g., to what extent we followed the agenda, or I found the session helpful).	0	1	2	3	4
24. My therapist and I agreed on therapy goals at the beginning of therapy (i.e., what I wanted to achieve in therapy).	0	1	2	3	4
25. My therapist and I evaluated the extent to which I had achieved my goals for therapy.	0	1	2	3	4
26. My therapist and I created a maintenance and relapse prevention plan to use following therapy completion	0	1	2	3	4

Therapist Adherence to Cognitive-Behavioural Therapy – Therapist version

The following statements describe activities which may have occurred in the therapy you have just delivered. Please rate how well each statement applies to the therapy using the scale below. Base your ratings on the actual occurrence of your activities, not your intentions. Please choose only one response per item. There are no right or wrong answers.

		Not at all	To a small degree	To a moderate degree	To a high degree	To a very high degree
1.	At the start of sessions, my patient and I went over how the session should be spent (an agenda or session plan).	0	1	2	3	4
2.	The patient and I spent the session on what we had agreed upon at the start of the session.	0	1	2	3	4
3.	The patient and I agreed on homework between sessions (e.g., practicing behaviours or recording thoughts, emotions, or events).	0	1	2	3	4
4.	The patient and I went over their homework since last session (e.g., exercises or recordings).	0	1	2	3	4
5.	I described why we focused on certain topics or exercises (i.e., provided a rationale).	0	1	2	3	4
6.	I explained how the patient's thoughts, emotions, and behaviours influence each other.	0	1	2	3	4
7.	The patient and I explored triggers to their problematic thoughts, emotions, and behaviours.	0	1	2	3	4
8.	The patient and I explored the origins of their problems and what maintains them.	0	1	2	3	4
9.	The patient and I explored the consequences of their behaviours.	0	1	2	3	4
10.	The patient and I explored how they view their problems.	0	1	2	3	4
11.	The patient and I planned how they can change their behaviours between sessions (e.g., doing things they fear, being more active, meeting people, or reducing control).	0	1	2	3	4
12.	In sessions, the patient did things they find uncomfortable or avoid (e.g., exposing themselves to objects, people, situations, or memories).	0	1	2	3	4
13.	The patient and I practiced new skills (e.g., being assertive, breathing training, or how to act in social situations).	0	1	2	3	4
14.	The patient and I role-played or rehearsed certain situations or behaviours together.	0	1	2	3	4
15.	The patient and I practiced finding alternative explanations to events or behaviours than those that first came to mind.	0	1	2	3	4
16.	The patient and I practiced noticing thinking errors or traps (e.g., black-and-white thinking, mind reading, or generalising).	0	1	2	3	4
17.	The patient and I explored how their problems are influenced by certain beliefs they have or rules they follow.	0	1	2	3	4
18.	The patient and I practiced challenging their thoughts or beliefs.	0	1	2	3	4
19.	The patient and I did exercises to test if their thoughts or beliefs were true.	0	1	2	3	4
20.	The patient and I practiced formulating new, more realistic thoughts or beliefs.	0	1	2	3	4
21.	The patient and I practiced solving problems.	0	1	2	3	4
22.	The patient and I repeatedly summarised our work during sessions.	0	1	2	3	4
23.	The patient and I evaluated the session at the end of sessions (e.g., to what extent we followed the agenda, or the patient found the session helpful).	0	1	2	3	4
24.	The patient and I agreed on therapy goals at the beginning of therapy (i.e., what the patient wanted to achieve in therapy).	0	1	2	3	4
25.	The patient and I evaluated the extent to which they had achieved their goals for therapy.	0	1	2	3	4
26.	The patient and I created a maintenance and relapse prevention plan to use following therapy completion.	0	1	2	3	4

Therapist Adherence to Cognitive-Behavioural Therapy – Observer version

The following statements describe activities which may have occurred in the session you have just listened to. Please rate how well each statement applies to the session using the scale below. Base your ratings on the actual occurrence of the therapist's activities, not on your interpretation of intent or the skill with which they are carried out. Activities do not need to be named using CBT terms, i.e., words such as "agenda" or "exposure" do not need to be used. Read each statement carefully and provide only one answer per statement. For each item, there is a description of what the therapist needs to do to receive four points.

0 = Not at all. The therapist does not carry out the activity at all.
1 = To a small degree. The therapist carries out the activity incompletely, missing most essential parts of it.
2 = To a moderate degree. The therapist carries out the activity incompletely, missing some essential parts of it.
3 = To a high degree. The therapist carries out the activity completely, missing a few less essential parts of it.
4 = To a very high degree. The therapist carries out the activity completely, without missing any parts of it.

		0	1	2	3	4
1.	**The therapist went over the agenda at the beginning of the session.** For four points, the agenda should be appropriate for one session, the patient be invited to make suggestions, and it should include homework review, main session focus and the setting of new homework. Scores are assigned based on the information available. For example, if the therapist refers to a written agenda, but the agenda items do not appear on the audio-recorded session, one point is assigned.					
2.	**The therapist followed the agenda.** For four points, all agenda items should be completed. The therapist and patient may deliberately change the agenda during a session, when clinically relevant (e.g., prioritising an unforeseen crisis, in contrast to excessive small talk). When deviations are clearly communicated and clinically relevant, no points are deducted.					
3.	**The therapist and patient agreed on homework for the next session.** For four points, the therapist should agree with the patient on a concrete homework task, linked to the purpose of the session/treatment, and plan its implementation.					
4.	**The therapist reviewed homework for the session.** For four points, the review of homework should include all assignments, highlight the results or potential lessons learnt, and the patient should be encouraged to take an active role.					
5.	**The therapist described why they focused on certain topics or exercises (i.e., provided a rationale).** For four points, the therapist should provide a comprehensible rationale which justifies a specific CBT procedure or technique.					
6.	**The therapist explained or referred to how the patient's thoughts, emotions, and behaviours influence each other.** For four points, the therapist should present a CBT model which clarifies the relationship between the patient's thoughts, emotions, and behaviours.					
7.	**The therapist and patient explored triggers or antecedents of problematic thoughts, emotions, or behaviours.** For four points, the therapist should identify triggers/antecedents and clarify their relationship to the patient's problematic thoughts, emotions, or behaviours.					
8.	**The therapist and patient explored the origins of the patient's problems or what maintains them.** For four points, the therapist should present a CBT model which explains the origins or maintenance of the patient's problems and make sure the patient understands.					
9.	**The therapist and patient explored the consequences of the patient's behaviour.** For four points, the therapist should identify the short- and long-term consequences.					
10.	**The therapist and patient planned how the patient can change their behaviour between sessions (i.e., planning exposure or behavioural activation).** For four points, the activities should be agreed upon, planned out so the patient knows how to perform them, and be related to the purpose of the session/treatment.					

11.	**The therapist supported the patient to do things in-session that they find uncomfortable or avoid (i.e., exposure).** For four points, exposure should have a clear goal, the therapist should enquire about the patient's experience (e.g., anxiety level), include approaching the phobic stimulus (e.g., object, person, situation, or traumatic memory) with some encouragement, reduce avoidance and/or safety behaviours, and be followed by evaluation of goals and lessons learned.	0	1	2	3	4
12.	**The therapist and patient practiced new skills (e.g., assertiveness, breathing or how to act in social situations, i.e., skills training).** For four points, skills training in-session should include concrete instructions, patient practice, and the therapist should enquire about results or potential lessons learnt.	0	1	2	3	4
13.	**The therapist and patient role-played situations or behaviours.** For four points, in-session role-play should include concrete instructions, enactment, and the therapist should enquire about results or potential lessons learnt.	0	1	2	3	4
14.	**The therapist and patient practiced finding alternative explanations for events or behaviours than those that first came to the patient's mind.** For four points, the therapist and patient should formulate several alternative thoughts or explanations for events or behaviours.	0	1	2	3	4
15.	**The therapist and patient practiced noticing thinking errors or thinking traps (e.g., "black and white thinking").** For four points, the therapist should describe and name several thinking traps, explain their function and potential consequences, and help the patient connect the traps to their thoughts.	0	1	2	3	4
16.	**The therapist and patient explored how the patient's problems are influenced by their automatic thoughts, beliefs, or rules.** For four points, the therapist and patient should identify the patient's automatic thoughts, beliefs, schemas, or rules, and the extent to which they are helpful to the patient.	0	1	2	3	4
17.	**The therapist and patient challenged thoughts or beliefs (e.g., evidence for and against).** For four points, the therapist should support the patient in defining an automatic thought, estimating how much they believe in it, disputing the thought (e.g., through evidence for and against) and then re-estimating how much they believe in the thought.	0	1	2	3	4
18.	**The therapist and patient did exercises to test if thoughts and beliefs were true (i.e., behavioural experiments).** For four points, the therapist and the patient should define a thought, and design and conduct a behavioural experiment to test the plausibility of the thought based on the results, while the therapist conveys a scientifically exploratory approach.	0	1	2	3	4
19.	**The therapist and patient practiced formulating new, more realistic thoughts or beliefs.** For four points, the therapist and patient should formulate new, more realistic thoughts or beliefs, in line with the purpose for the session/treatment.	0	1	2	3	4
20.	**The therapist supported the patient in finding their own solutions to their problems (i.e., problem solving as a structured intervention).** For four points, problem solving should be a step-by-step process including a clear problem formulation, brainstorming possible solutions, identifying advantages and disadvantages of these solutions, selecting the best available alternative, and planning its implementation.	0	1	2	3	4
21.	**The therapist and patient repeatedly summarised their work in session.** For four points, both regular summaries after completed parts of the session and a full summary at the end of the session are required.	0	1	2	3	4
22.	**The therapist and patient evaluated the session.** For four points, the evaluation must cover several aspects of the session, such as the extent to which they followed the agenda, whether the patient found the session helpful, the patient's reactions to the therapist's behaviours, or lessons learnt.	0	1	2	3	4
